# A Post-Developmental Genetic Screen for Zebrafish Models of Inherited Liver Disease

**DOI:** 10.1371/journal.pone.0125980

**Published:** 2015-05-07

**Authors:** Seok-Hyung Kim, Shu-Yu Wu, Jeong-In Baek, Soo Young Choi, Yanhui Su, Charles R. Flynn, Joshua T. Gamse, Kevin C. Ess, Gary Hardiman, Joshua H. Lipschutz, Naji N. Abumrad, Don C. Rockey

**Affiliations:** 1 Department of Medicine, Medical University of South Carolina, Charleston, SC, 29425, the United States of America; 2 Department of Biology, Vanderbilt University, Nashville, TN, 37232, the United States of America; 3 Department of Surgery, Vanderbilt University, Nashville, TN, 37232, the United States of America; 4 Department of Pediatrics, Vanderbilt University, Nashville, TN, 37232, the United States of America; 5 Department of Medicine, Ralph H. Johnson Veterans Affairs Medical Center, Charleston, SC, 29401, the United States of America; National University of Singapore, SINGAPORE

## Abstract

Nonalcoholic fatty liver disease (NAFLD) is one of the most common causes of chronic liver disease such as simple steatosis, nonalcoholic steatohepatitis (NASH), cirrhosis and fibrosis. However, the molecular pathogenesis and genetic variations causing NAFLD are poorly understood. The high prevalence and incidence of NAFLD suggests that genetic variations on a large number of genes might be involved in NAFLD. To identify genetic variants causing inherited liver disease, we used zebrafish as a model system for a large-scale mutant screen, and adopted a whole genome sequencing approach for rapid identification of mutated genes found in our screen. Here, we report on a forward genetic screen of ENU mutagenized zebrafish. From 250 F2 lines of ENU mutagenized zebrafish during post-developmental stages (5 to 8 days post fertilization), we identified 19 unique mutant zebrafish lines displaying visual evidence of hepatomegaly and/or steatosis with no developmental defects. Histological analysis of mutants revealed several specific phenotypes, including common steatosis, micro/macrovesicular steatosis, hepatomegaly, ballooning, and acute hepatocellular necrosis. This work has identified multiple post-developmental mutants and establishes zebrafish as a novel animal model for post-developmental inherited liver disease.

## Introduction

In order to study mechanisms of inherited liver disease progression, and to develop effective treatments for individuals with different mutations, it is essential to have appropriate models. Forward and reverse genetic screens in zebrafish have proven to be powerful in the study of developmental defects. Of note, studies done focusing on metabolic disorders, including inherited liver diseases, have largely not been performed. Many genes and signaling pathways controlling metabolic pathways and liver disease in mammals are highly conserved in zebrafish [1,2], though, again, few known metabolic mutants have been discovered from forward genetic screening. Recent studies in *lkb1* and *tsc2* mutant zebrafish, found using a reverse genetic approach, show elevated cellular metabolic activities, and post-developmental liver defects at 7 days post fertilization (dpf) including steatosis and hepatomegaly (normal liver development is complete by 5 dpf) [3,4]. In addition to the *lkb1* and *tsc2* mutants, CDP-diacylglycerol-inositol 3-phosphatidyltransferase (*cdipt*) mutants from a forward genetic screen were recently reported [5]. The *cdipt* mutants demonstrate various features of liver diseases, including, steatosis, ballooning of hepatocytes and cell death in the liver. Interestingly, all three mutants did not show significant phenotypic defects at 5 dpf. Those results suggested to us that novel mutants with liver defects could be found in post-developmental stages beyond 5 dpf [1].

Because most mutant screening in zebrafish focused on early developments, we believe that this accounts for the relatively few post-developmental liver disease models found to date in zebrafish. There was one liver mutant screen done at 5 dpf stage to identify mutations involved in hepatic outgrowth. Those mutants from that screen showed pathophysiological liver defects such as steatosis and/or hepatomegaly; however, they also exhibited developmental defects such as jaw defects and/or smaller intestine [6]. Of note, the liver mutants from previous screening did not recapitulate hepatic injury such as ballooning of hepatocytes or hepatic cell death, which can be observed in advanced liver disease in humans, such as steatohepatitis or cirrhosis.

To identify novel inherited liver diseases and to help establish models in zebrafish, we performed a screen for genetic mutations causing liver abnormalities such as hepatomegaly and/or steatosis during post-developmental stages using forward genetic screening of ethyl-N-nitrosourea (ENU)-mutagenized zebrafish. We identified 19 unique mutant zebrafish lines showing hepatomegaly and steatosis, without developmental defects, from 250 F2 families of ENU mutagenized zebrafish. These mutants exhibited various liver disease phenotypes, ranging from mild steatosis to acute hepatocellular necrosis.

## Materials and Methods

### Ethics Statement

Zebrafish studies were approved by the Vanderbilt University Animal Care and Use program (Animal study protocol number; M/09/401) and Medical University of South Carolina Division of Laboratory Animal Resources (Animal study protocol number; AR3364)

### Fish husbandry

The zebrafish strain used in this study was AB*. Adult were maintained at 28.5°C and fed twice a day. Embryos were obtained from natural mating and raised at 28.5°C in egg water (0.3mg of sea salt/L). All of mutants lines were outcrossed with wild type AB* at least three times to reduce additional background mutations.

### N-ethyl-N-nitrosourea (ENU) mutagenesis

ENU treatment was performed following the procedures as described with few modifications (https://wiki.zfin.org/display/prot/ENU+Mutagenesis+Procedure+With+Increased+Fish+Survival). 4–8 month old AB* males were incubated for 1 hour in a solution of 3.5 mM ENU four times at intervals of 7 days. ENU treatment was performed at 20°C in the hood and zebrafish were transferred to a recovery tank (19°C, 10mg/ml MESAB). ENU treated males were outcrossed with Tg(foxd3:GFP) to generate F2 lines. This liver screening project was performed as a joint project with a brain laterality mutant (pineal) screen, which was done at 3 dpf. After screening of pineal mutants, embryos were rinsed once and harvested at 8 dpf. Larvae were monitored once at 5 dpf and mutants with developmental defects were excluded from this screening. A total of 250 F2 lines, from 5 dpf to 8 dpf, were used for the screening of post-developmental liver- defects: steatosis and/or hepatomegaly.

### Oil Red O (ORO) Staining of Whole Mount Zebrafish

For whole mount staining at the larval stage, larvae were fixed in 4% PFA overnight. The same numbers of control and mutant larvae (5 to 10 larvae each) were transferred to 1.5 mL Eppendorf tube and rinsed three times (5 minutes each) with 1X PBS/0.5% Tween-20 (PBS-Tween). After removing PBS-Tween, larvae were stained with a mixture of 300 μL of 0.5% ORO in 100% isopropyl alcohol and 200 μL of distilled water for 15 minutes. Larvae were then rinsed three times with 1X PBS-Tween, and twice in 60% isopropyl alcohol for 5 minutes each. Finally, they were briefly rinsed in PBS-Tween and fixed in 4% PFA for 10 minutes. Larvae were mounted in glycerol prior to imaging.

### Liver Histology

Embryos were fixed in 4% paraformaldehyde from overnight to two days at 4°C. Fixed embryos were embedded in 1.2% agarose/5% sucrose, and saturated in 30% sucrose at 4°C for 1 to 2 days. Blocks were frozen on the surface of 2-methyl butane chilled using liquid nitrogen. 10 μm sections were collected on microscope slides using a Leica cryostat. Sections were kept in -80°C before use.

### H&E

To avoid staining variation, 3 control and 3 mutant larvae were processed together in the same slide glass and all of staining were performed at least 3 times. Slides were processed in a Sequenza Slide Rack. The H &E stain was conducted in the Translational Pathology Core laboratory at Vanderbilt University using a DAKO Artisan Link Staining System.

### Oil Red O (ORO)

For ORO staining in sectioned larvae, 150 μm of fresh ORO (a mixture of 300 μL of 0.5% ORO in 100% isopropyl alcohol and 200 μL of distilled water) were dropped on the slides and stained for 30 secs. Washed with tap water and mounted with 100% glycerol.

### Filipin

For free cholesterol staining of transversely sectioned larvae, slides were soaked with 1X PBS for 5 minutes, then Filipin complex diluted 1:500 (Sigma, F-976) was added directly to the slides and allowed to stain for 1 minute in the dark. Slides were washed with PBS and mounted with 75% glycerol. Images were taken using the DAPI channel of a fluorescent microscope.

### DAPI

Sections were rehydrated in 1x PBS and mounted in Vectashield with DAPI (Vector laboratories). Images were acquired using a Zeiss Axiovert 200M microscope with Zeiss AxioCam MRm and Hamamathu digital cameras. Digital images were processed using Adobe Photoshop CS5 and Adobe illustrator CS5. All images received only minor modifications, with control and mutant sections always processed in parallel.

### Whole genome sequencing

Genomic DNA from 10 normal siblings and 10 homozygous mutants were used as template DNAs for whole genome sequencing. The Vanderbilt Sequencing Core for *mu110* mutant line and Medical University Sequencing Core for *mu107* and *mu108* mutant lines performed sequencing of samples using an Illumina HiSeq200 Platform with 100bp paired-end reads, resulting in approximately 10 fold genomic coverage. The sequencing results were uploaded to the SNPtrack Mapping server (http://genetics.bwh.harvard.edu/snptrack/), and mapped mutations re-confirmed by sequencing and genotyping of individual mutants.

### Data Accession

The whole genome sequencing reads have been deposited in the NCBI Short Read Archive (SRA) database under the accession numbers SRR1826582, SRR1826622, SRR1826582 and SRR1826622.

## Results

### Genetic Screen for post-developmental liver disease identified 19 novel mutants in zebrafish

ENU treatment generates random point mutations throughout the genome of spermatogonia in zebrafish males, and has been widely used for forward genetic screening of mutants in zebrafish [7]. Despite several thousands of mutants having been identified using forward genetic screening during development, less than 20 mutants with liver defects have been uncovered to date but mostly involved in liver specification during development [1]. Analysis of the published mutants reveals conserved function of genes involved in liver development between mice and zebrafish, though the mutations were mostly involved in liver specification and few were involved in liver outgrowth. Very few, if any, mutants showing post-developmental liver disorders were discovered. The vast majority of investigators in the zebrafish field have focused on discovering mutants before 5 dpf when larvae start to eat independently, implying the developmental processes are complete, and have not considered post-developmental disorders such as physiological liver diseases. We previously found that homozygous *tsc2* mutants exhibit hepatomegaly at 7 dpf, but they do not develop any obvious defects prior to this time [4]. We, therefore, hypothesized that many mutants with post-developmental liver phenotypes, such as steatosis, hepatomegaly and liver injury, can be screened for after liver outgrowth is complete at 5 dpf.

**Fig 1:**
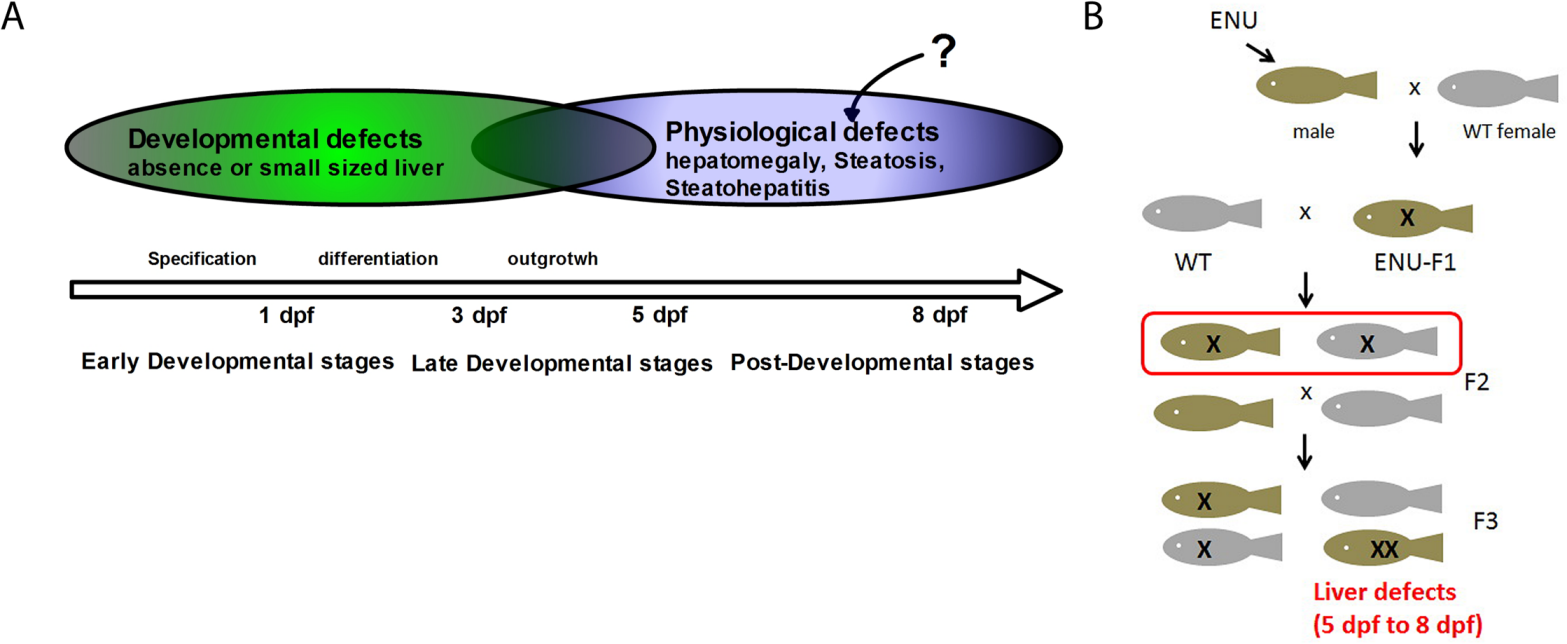
Strategy of forward genetic screening to identify novel IEM models with liver phenotypes. In (A) is depicted the strategy to identify mutants with liver defects during post-developmental stages in zebrafish. In (B) is shown a schematic diagram of forward genetic screening to identify inherited liver disease models. The specific screening criteria involved identification of offspring without developmental defects that had hepatomegaly and/or hepatic steatosis.

**Fig 2:**
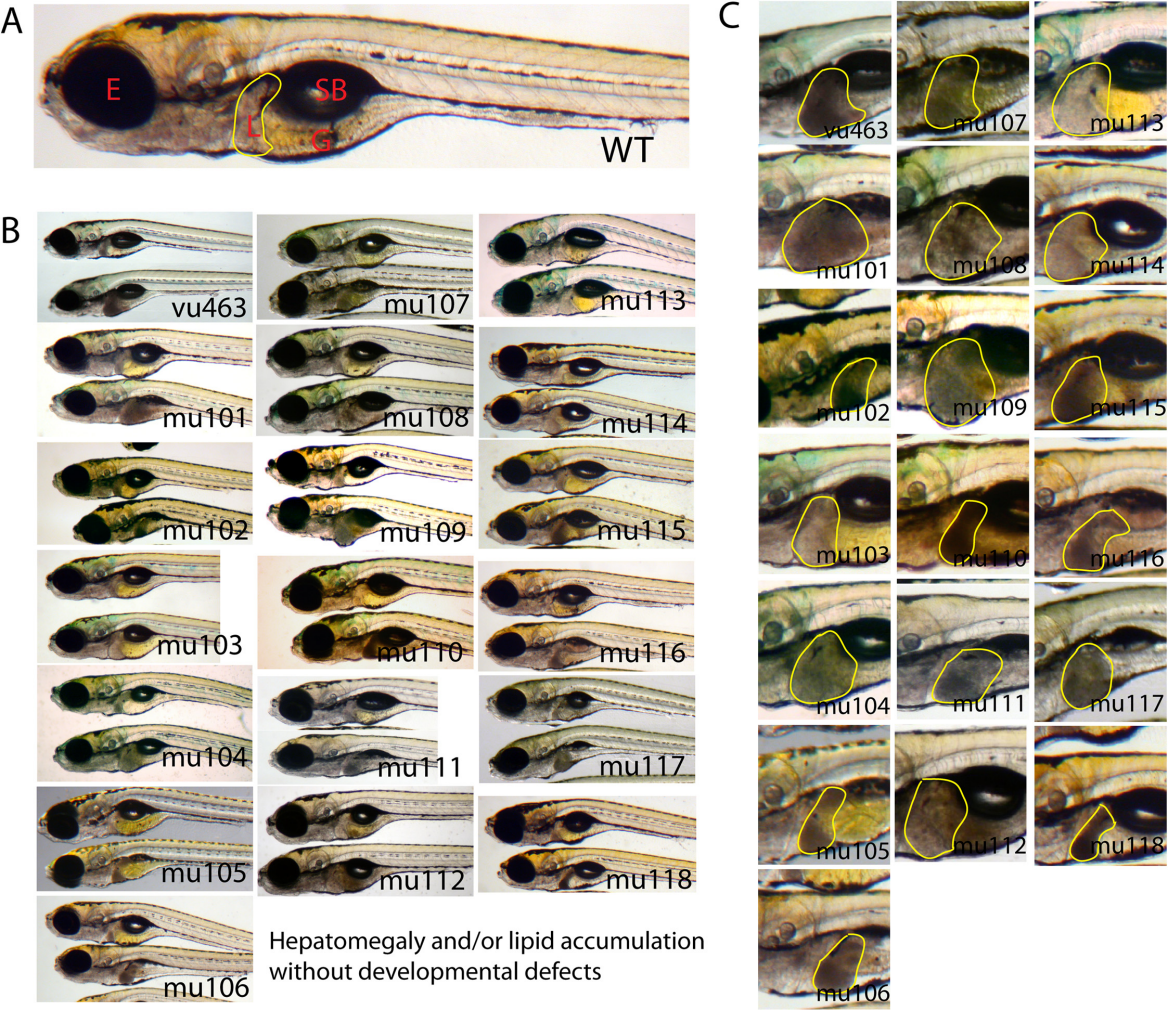
Zebrafish mutants exhibit post-developmental liver defects without developmental phenotypes. (A) A wild type control at 7 dpf. Livers are outlined with yellow line. (B) Control siblings are on the top and homozygous mutants are on the bottom of each panel. (C) Magnified images of livers (outlined with yellow) in homozygous mutants. E = eye, L = liver, G = gut and SB = swim bladder. “mu”, stands for MUSC, followed by a number that designates the unique mutants identified in the screen. n>100 per each mutant.

To generate a mutant library with a high frequency of mutations throughout the genome, 4–8 month old AB* males were incubated for 1 hour in a solution of 3.5 mM ENU four times at intervals of 7 days. Mutagenized F0 male fish were outcrossed with wild type female fish to generate F1 lines. Individual F1 fish were outcrossed again with wild-type zebrafish to generate F2 families. We performed screening of 250 F2 lines by incrossing within the family to obtain homozygous F3 mutant larvae from 5 to 8 dpf, following completion of hepatic outgrowth (Fig 1). Since zebrafish larvae are still transparent at a juvenile stage, we used a stereomicroscope to identify liver defects. We identified 19 novel mutant zebrafish lines out of 250 lines showing obvious liver defects such as hepatomegaly and/or a dark-colored liver, which suggests liver steatosis (Fig 1). None of the mutants display liver specification defects or other obvious morphological defects and growth retardation compared to control siblings (Fig 2). Three of the mutants have a hepatomegaly phenotype without color changes in the liver (*7048* ^*mu103*^, *7604a* ^*mu113*^,*7639* ^*mu114*^), 11 of the mutants have hepatomegaly and a dark-colored liver (electron transfer flavoprotein a (*etfa)* ^*vu463*^, *6977a* ^*mu101*^, *7521* ^*mu104*^, *7610* ^*mu106*^, *7653* ^*mu107*^, *7703* ^*mu108*^, *2711b* ^*mu109*^, *tsc2* ^*mu111*^,*6977c* ^*mu112*^, *7043* ^*mu115*^, *7604c* ^*mu117*^), and 5 mutants have a dark-colored liver phenotype without hepatomegaly (*6977b* ^*mu102*^, *7600* ^*mu105*^, *7466* ^*mu110*^,*7406* ^*mu116*^, *7641* ^*mu118*^). None of the mutants identified had obvious developmental defects in body shape, growth retardation and craniofacial defects that are commonly observed in forward genetic screens in zebrafish [7].

### Liver mutants exhibit hepatomegaly alone, steatosis only or hepatomegaly and steatosis

**Fig 3:**
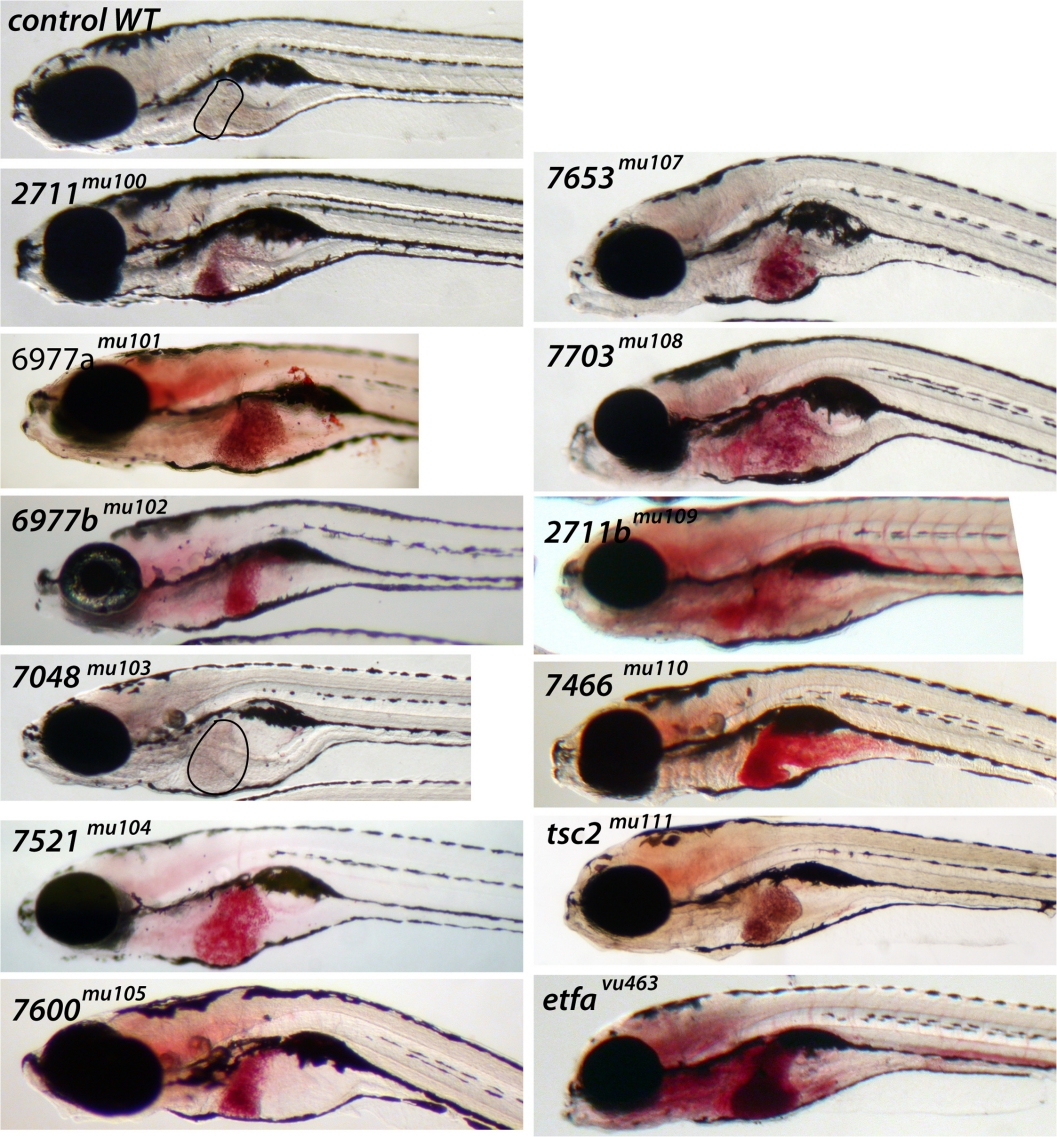
Whole mount Oil Red O (ORO) staining in representative mutants. A control, wild type example, at 8 dpf, is shown in top of left panel. A sample of 11 representative mutants with a dark-colored liver and one mutant with clear liver (*7048* ^*mu103*^) were subjected to ORO staining. Clear colored livers in control and *7048* ^*mu103*^ are outlined with black. n = 9/9 per each mutant.

To investigate the observation that dark colored livers may contain lipid droplets, we performed whole mount ORO staining of screened mutants (Figs 2 and 3). All 16 mutants with a dark-colored liver (Fig 2) exhibited intense ORO staining in the liver. In contrast, there was no significant ORO staining in 3 mutants with a transparent liver (not shown). Thus, these data indicate that the darkish liver can be useful marker for rapid screening of mutant with steatosis in the future.

**Fig 4:**
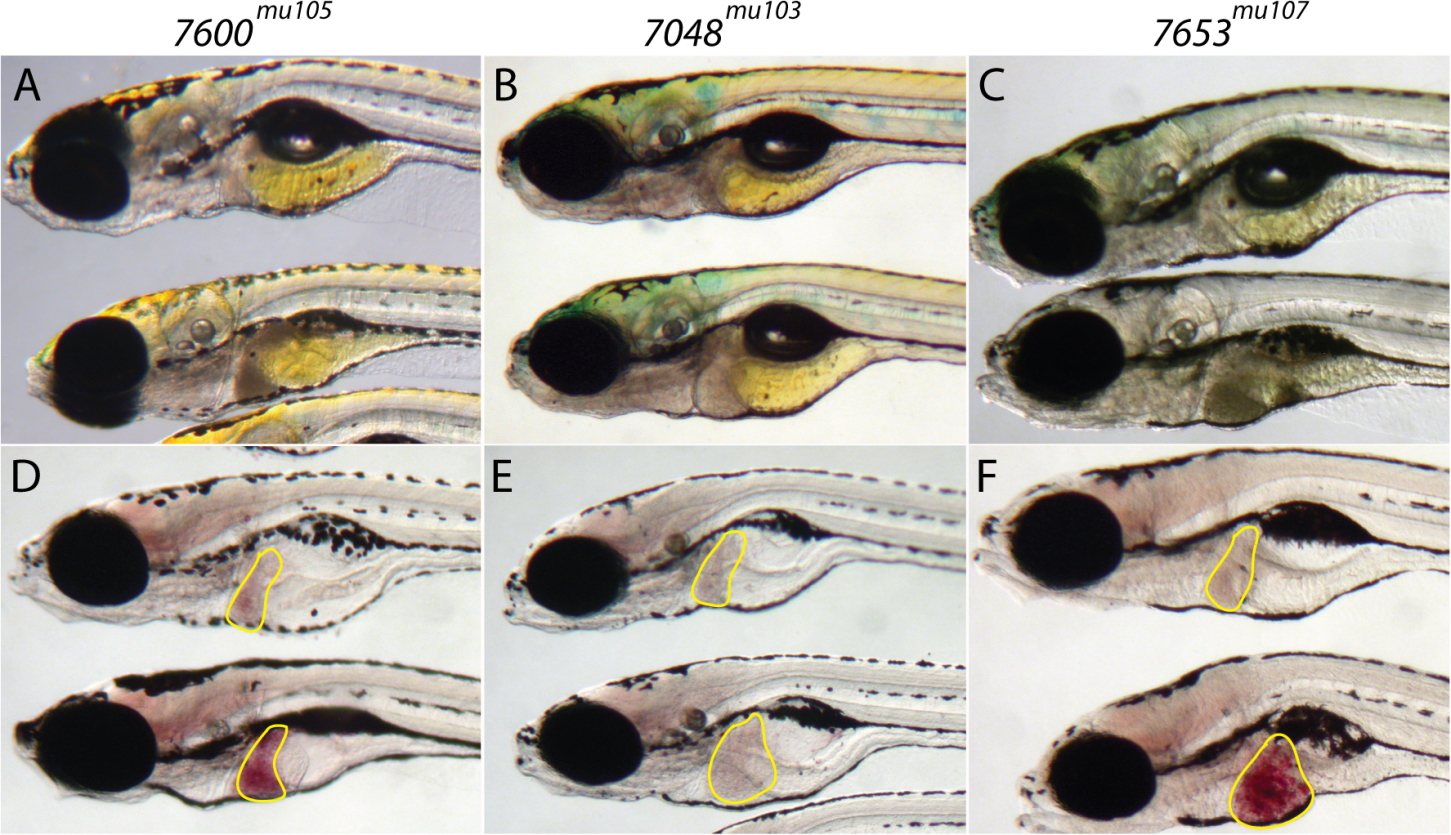
Three liver phenotypes were identified in the forward genetic screen. In all panels, the normal sibling is on top and the mutant is on the bottom. On the left, in (A, D) is shown a representative example of a mutant with hepatomegaly alone (n = 9/9). In (B, E) is shown a representative mutant with steatosis alone (n = 9/9), and (C,F) show a mutant with both hepatomegaly and steatosis (n = 9/9). In (A-C), are depicted representative stereomicroscopic images and in (D-F), whole mount ORO stained specimens. The liver is outlined in yellow.

**Fig 5:**
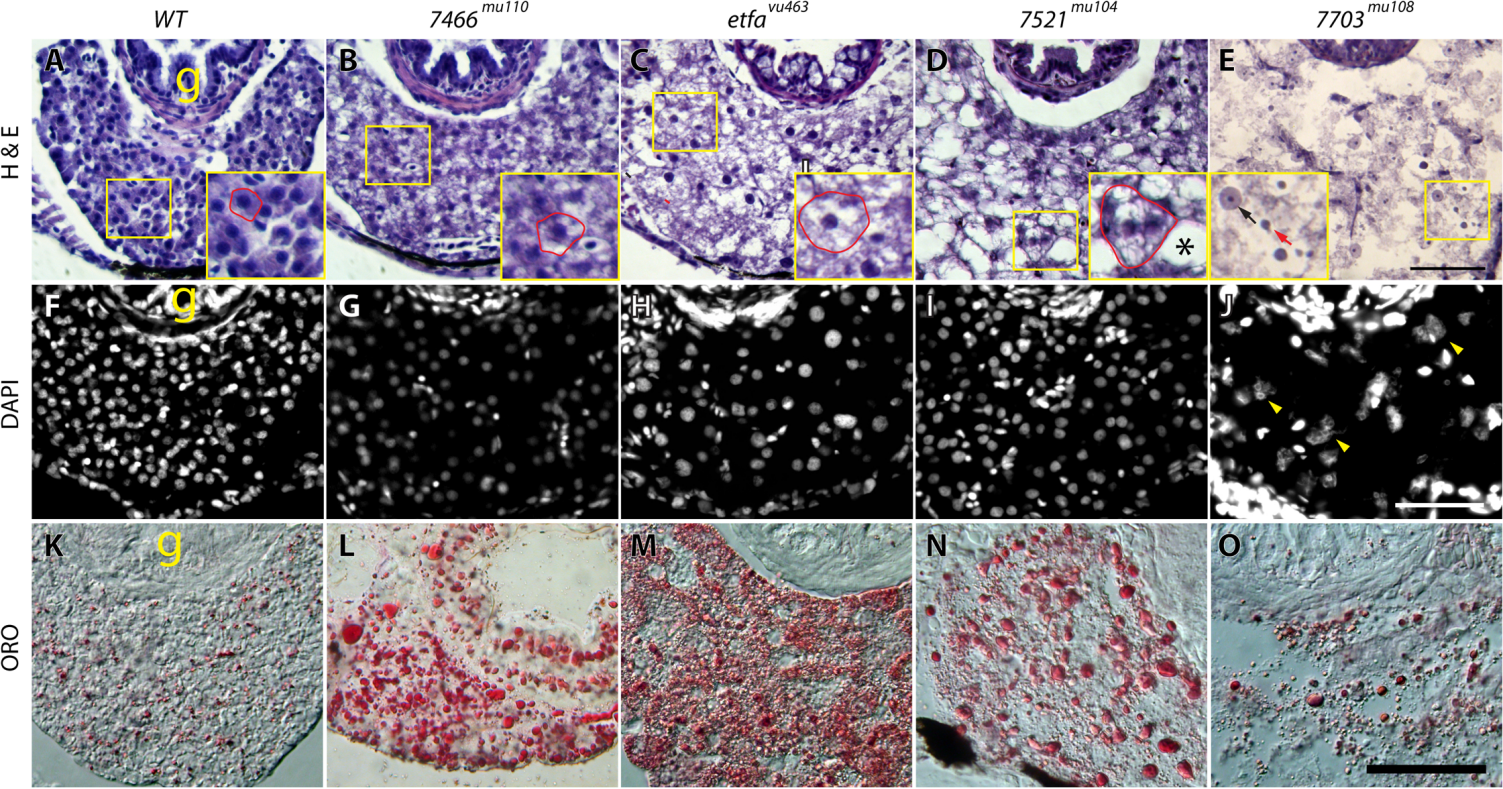
Histological phenotypes. On the left panel, H & E (top), DAPI (middle) and oil red oil (ORO, bottom) stained zebrafish livers at 8 dpf are shown. Wild-type control (A,F,K), and mutants (B-E, G-J, L-O) are shown. (B) shows and example of microvesicles in hepatocytes, (C) depicts a liver with swollen hepatocytes, (D) shows a liver with accumulation of large vesicles, and prenecrotic hepatocytes (asterisk), while (E) shows hepatic lysis. The black arrow in (E) points to a nucleus with nuclear membrane and the red arrow points to a condensed nucleus without nuclear membrane. The yellow arrowheads in (J) indicate granulated nuclei. g = gut. Scale bar = 100 μm (A-E) and 50 μm (F-O). n = 9/9 per control and each mutant.

We identified 3 major phenotypes including hepatomegaly alone, steatosis only, and hepatomegaly and steatosis (Fig 4). *7600* ^*mu105*^ mutants exhibit steatosis with a normal sized liver (Fig 4A and 4D), *7048* ^*mu103*^ mutants have hepatomegaly, but no increase in lipid accumulation (Fig 4B and 4E), and *7653* ^*mu107*^ mutants show significant hepatomegaly and steatosis compared to control siblings (Fig 4C and 4F). In addition to lipid accumulation in the liver, histological analyses of each mutant showed various degrees of liver injury including: accumulation of microvesicules in hepatocytes (*7466* ^*mu110*^, Fig 5B), ballooning of hepatocytes (*etfa* ^*vu463*^, Fig 5C), severe ballooning of hepatocytes with macrovesicles (*7521* ^*mu104*^, Fig 5D) and acute hepatocyte necrosis (*7703* ^*mu108*^, Fig 5E). We also observed increase of hepatocyte size in *7466* ^*mu110*^, *etfa* ^*vu463*^, *7521* ^*mu104*^ mutant livers.

Interestingly, *7703* ^*mu108*^ showed liver cell death, and occurred within 2 days following the increase in liver size at 6 dpf (not shown). H & E and DAPI staining in the *7703* ^*mu108*^ liver shows loss of nuclei membrane and granulation, implying hepatocyte karyorrhexis (Fig 5E and 5J). Histological analyses are shown in Table 1. Interestingly, we found that *7604c* ^*mu117*^ and *7703* ^*mu108*^ mutants developed hyperlipidemia (S1C and S1D Fig) in a manner similar to the *etfa* ^*vu463*^ mutant, which involves lipid β-oxidation, suggesting that these mutants may also carry mutations in genes involved in lipid metabolism. Seven mutants showed ballooning of hepatocytes, which may be a marker for hepatocyte injury. Two mutants showed severe hepatocyte cell necrosis, identified within 2 days following identification of hepatomegaly.

**Table 1:** Pathological defects in mutants.

Genes	Allele	Mutated site	Steatosis (ORO)	Hyperlipidemia	Hepatomegaly (H E)	Ballooning hepatocytes (H E)	Hepatic cell death (DAPI)
				in blood (ORO)			
n.d.	mu100	n.d.	+	-	+	-	-
n.d.	mu101	n.d.	++	-	++	+++	+++
n.d.	mu102	n.d.	++	-	+	-	-
n.d.	mu103	n.d.	-	-	+	n.d.	-
n.d.	mu104	n.d.	++	-	++	+++	-
n.d.	mu105	n.d.	++	-	-	-	-
n.d.	mu106	n.d.	n.d.	n.d.	+	+++	n.d.
**lyst**	mu107	Q336->stop	++	-	+	-	-
**apoa1bp**	mu108	R26->stop	+	+	++	+++	+++
n.d.	mu109	n.d.	-	-	+++	+++	-
**vmp1**	mu110	exon11/intron	+++	-	-	-	-
**tsc2**	mu111	R25->stop	+	-	+	++	-
**etfa**	vu463	G290->stop	+++	+	++	++	-

An analysis of the 13 mutants analyzed is shown; liver steatosis, hyperlipidemia in the blood, hepatomegaly, hepatocyte injury (ballooning and hepatic cell death) were graded according to the following scale (changes compared to control siblings for each category):- = no change, + = mild, ++ = moderate, +++ = severe changes.

### Complementation testing results confirmed diversity of mutated genes causing liver disease

To test whether there are redundant mutations in a single gene that might produce similar liver defects, we did a complementation test by crossing each mutant line with the rest of mutants. Complementation testing confirmed that each individual mutant did not complement the other mutants in the screen, suggesting all of the mutant lines are unique (not shown). However, the *mu111* mutant resembled the *tsc2* ^*vu242*^ mutant that we had found previously using a reverse genetic screening approach [4]. Complementation testing with heterozygous *tsc2* ^*vu242*^ confirmed that *mu111* carries a C to T mutation at 733bp in *tsc2* gene and the nonsense mutation causing R245 to stop in the tsc2 confirmed by sequencing of mutant larvae (not shown).

### High-throughput mapping by whole genome sequencing

**Fig 6:**
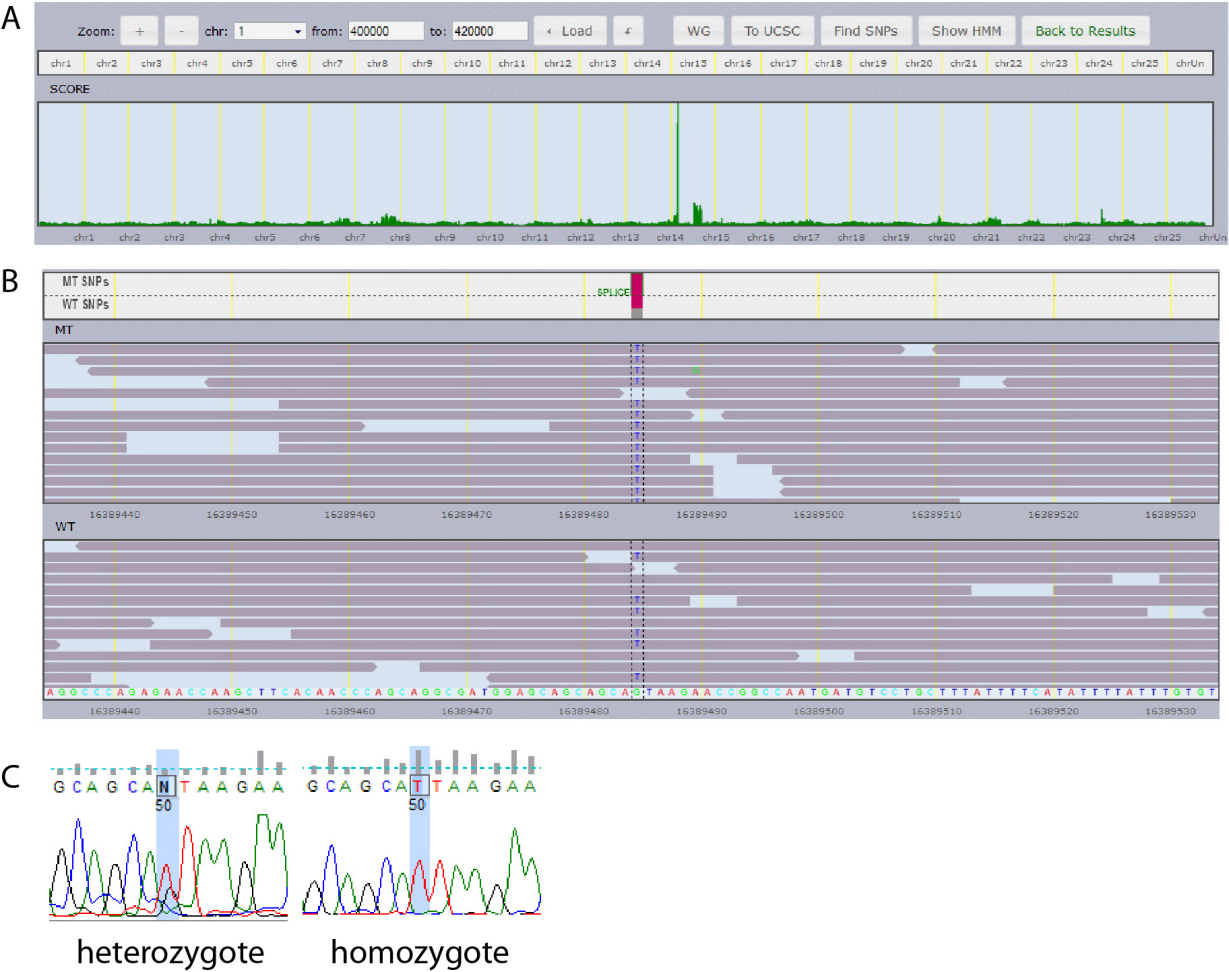
SNPTrack analysis and sequencing of heterozygous and homozygous *vmp1* ^*mu110*^ mutant. In (A) is depicted a distribution of SNPs in whole genome, implying that the highest probability of linked mutation in mu110 phenotype. (B) Homozygous mutant pool carries G->T conversion in vmp1. (C) Confirmation of the G to T mutation in vacuole membrane protein 1 (vmp1) from DNAs of a heterozygous and homozygous *7466* ^*mu110*^ mutant larvae.

Rapid identification of mutated genes is essential in to identify large number of mutants from a forward genetic screen in ENU-induced zebrafish mutants. Traditional mapping by PCR based approaches, using various polymorphic marker primers, is time and labor intensive to identify mutated locus in a single mutant zebrafish. However, recent technical advances in high-throughput sequencing allowed us to sequence the entire zebrafish genome to directly identify mutations in multiple mutants at the same time within weeks [8]. The technique has been validated by us previously to identify a mutation in *etfa* as causative mutation in the *vu463* allele [9]. We also found a mutation in splicing donor site between exon 11 and adjacent intron region of *vacuole membrane protein 1* (*vmp1*) in *7466* ^*mu110*^ mutant by whole genome sequencing/SNPtrack (Fig 6). In addition, we recently identified two more non-sense mutations in *mu107* and *mu108* mutant alleles. The 7653^*mu107*^ mutant carries C to T mutation at 987bp in lysosomal trafficking regulator (*lyst*) and the nonsense mutation causes Q336 to stop in the lyst. C to T conversion in at apolipoprotein a1 binding protein (*apoa1bp*) causes a nonsense mutation at R26 position of apoa1bp in *7703* ^*mu108*^ (S2 Fig).

## Discussion

To better understand molecular mechanisms of various liver diseases, knockout and gain of function rodent models have been established [10–13]. However, a number of these genetic models do not appear to exhibit consistent pathological and metabolic contexts. To further expand an understanding of genetics in liver disease, forward genetic screening of randomly mutagenized animal models provides a powerful tool to identify important genetic variants that can cause liver disease. Over the past two decades, zebrafish have been extensively used to study developmental processes and have led to a clearer understanding of the signaling networks important in fate specification, differentiation and proliferation of hepatic progenitors (reviewed in [1]). To our knowledge, the current study is the first successful forward genetic screen at post-developmental stage 5 to 8 dpf in zebrafish. Moreover, these data provide confirmation that zebrafish provide a powerful model to study mechanisms of post-developmental liver disease [14–16] and validate zebrafish as a model system to study mechanisms of liver disease related cellular metabolism [4,8].

Here, we have identified 19 unique mutant zebrafish lines showing various degrees of liver defects, including the electron transfer flavoprotein a (*etfa)* mutant that we have previously reported [9]. The zebrafish glutaric acidemia type II (GA-II) model is caused by a nonsense mutation in *etfa*, which is a central protein in the mitochondrial β-oxidation pathway [9]. Importantly, the *etfa* zebrafish mutant strikingly recapitulates various pathological and biochemical symptoms observed in human GA-II patients, including steatosis and hepatomegaly due to mitochondrial defects.

Our high yield of post-developmental liver mutant screen (19/250 lines, 7.6%) resulting from mutations in different genes, which was confirmed by complementation testing, was notable. Previous attempts to identify mutants with liver defects by ENU-mutagenesis found only 4 mutants out of 2746 lines (0.15%) [17,18]. The fact that using a post-developmental approach to find inherited liver disease models such as we used led to an order of magnitude higher mutant screening rate implies that large-scale post-developmental genetic screening in zebrafish could introduce many more novel models in the field of liver disease.

Many inborn errors of metabolism (IEM) are known to cause liver abnormalities, including steatosis, hepatomegaly and hepatocyte injury as well as defects in other organs such as kidney and pancreas and brain resulting from the accumulation of toxic metabolites, alterations in metabolism, or organelle dysfunction. Although we do not show additional organ defects of screened mutants in this paper, we found at least 6 mutants exhibiting kidney tubule defect (*mu100*, *mu107*, *mu108*, *mu110*, *mu111* and *vu463*, data not shown). Thus, our liver mutant may have additional defects in other organs although it was not observed during mutant screening due to relatively mild symptom compared to liver defects. We are going to continue comprehensive studies for individual mutant lines in the future. Individuals with heterozygous mutation in our liver mutants may appear to be healthy, but may be vulnerable to stresses such as a western diet, obesity, alcohol consumption and/or exposure to hepatotoxic medicines, any of which may cause liver disease. A diverse array of genes directly involved in metabolic pathways, including those involved in carbohydrate, amino acid, fatty acid and even metal metabolism are commonly accepted as causative in IEM, but genes with indirect effects on metabolic pathways such as in autophagy, mechanistic target of rapamycin (mTOR) signaling and proteasomal degradation pathways have not been clearly studied in IEM. Since mutations in genes controlling these pathways show similar liver specific pathology (hepatomegaly, steatosis, hepatocyte injury), we speculate that underlying mechanisms regulating the functions of intracellular organelles including lysosomes, peroxisomes and mitochondria will be uncovered. In support of this concept, one of the zebrafish mutants identified here, namely the GA-II mutant, has already been shown to be a mutation directly regulating cellular metabolism [9]. In addition to the GA-II example of IEM, we show here that the *vmp1* mutation causes steatosis (*7466* ^*mu110*^ mutant, Fig 5L); there is no animal model of this mutation reported. Previous study showed that *vmp1* plays roles in inhibition of metastasis and proliferation of hepatocellular carcinoma [19] and an *in vitro* study showed that *vmp1* plays important role in autophagy processes [20]. The link between autophagy and steatosis is notable, as autophagy is well-known to be prominent in a variety of liver diseases associated with steatosis [21]. Thus, we speculate that *vmp1* may play an important role in mitochondrial function indirectly by controlling autophagy process. This is supported by previous work demonstrating that inhibition of autophagy in *etfa* mutants due to mTORC1 activation, which is another upstream signaling pathway in autophagy (not shown). Chediak-Higashi Syndrome (CHS) is one of IEM and an autosomal recessive lysosomal storage disorder that arises from a mutation of a lysosomal trafficking regulatory protein (*lyst*), which leads to a severe immunodeficiency, hypopigmentation and neurological symptoms in humans [22,23]. The disease is characterized by enlarged lysosomes, defective autophagy, and hepatosplenomegaly [24]. We noticed that the *lyst* ^*mu107*^ mutant showed pale skin tone, hepatomegaly, liver steatosis and kidney defects (not shown). Comprehensive analyses will be necessary to compare symptoms in CHS as follow up study. Thus, our novel mutant screening approach for post-developmental liver defect may identify novel IEM models too.

An important implication of our findings is that these novel liver disease models could be used for *in vivo* drug screening as zebrafish embryos are ideal to screen small molecules with therapeutic effects [25]. In addition, heterozygous adult mutants in large numbers can be exposed to high-fat and/or high-cholesterol diets, or alcohol to evaluate genetic susceptibility whether those mutations can increase liver disease occurrence.

In conclusion, mutants from our screen exhibited several core liver phenotypes, and identification of the mutated genes and characterization of the molecular pathways leading to liver disease in these mutants will provide important new information regarding pathways involved in inherited liver diseases. It is anticipated that identification of the pathways in which these molecules play a role by gene interactome analysis, will elucidate therapeutic targets in patients with liver disease.

## Supporting Information

S1 Fig Hyperlipidemia in *etfa* ^*vu463*^, *7604c* ^*mu110*^ and *7703* ^*mu108*^ mutants with steatosis. ORO staining in plasma of heart chamber in wild-type control (A), *etfa* ^*vu463*^ (B), *7604c* ^*mu110*^ (C) and *7703* ^*mu108*^ (D) mutants. A = Atrium, V = Ventricle. Scale bar = 50 μm.
(TIF)

S2 Fig Identification of mutated genes in *7653* ^*mu107*^ and *7703* ^*mu108*^. null mutations in *lyst* (A) and *apoa1bp* (B) were identified by whole genome sequencing and both mutations were confirmed in heterozygous mutant adult. C to T conversion caused Q336 to stop in lyst (A) and R26 to stop in apoa1bp (B).
(TIF)
