# Supplementary figures and images for: A Post-Developmental Genetic Screen for Zebrafish Models of Inherited Liver Disease
Source: PLoS One. 2015 May 7;10(5):e0125980. doi: 10.1371/journal.pone.0125980 (PMC4423964; doi:10.1371/journal.pone.0125980)

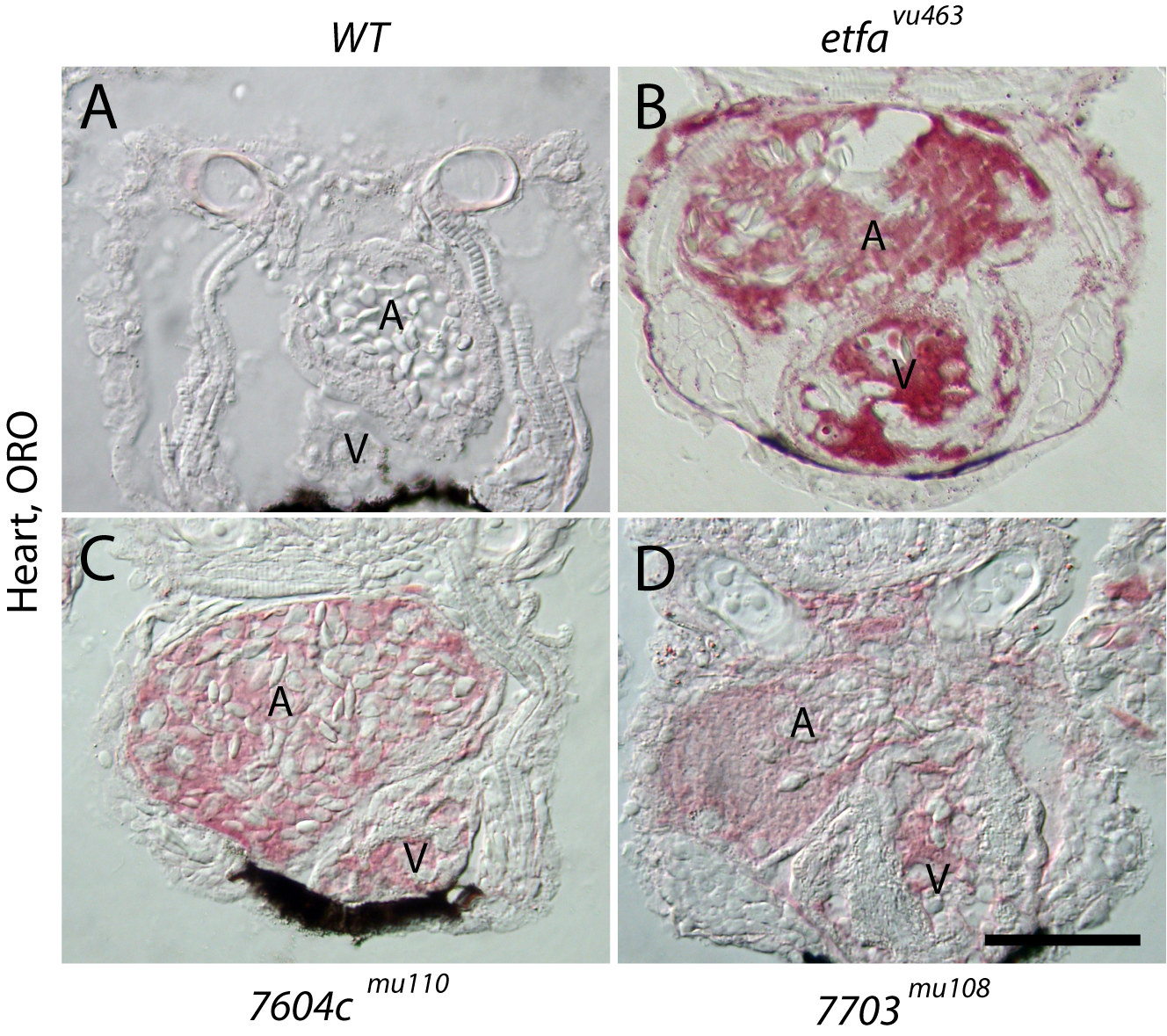

Supplement: S1 Fig — ORO staining in plasma of heart chamber in wild-type control (A), etfa vu463 (B), 7604c mu110 (C) and 7703 mu108 (D) mutants. A = Atrium, V = Ventricle. Scale bar = 50 μm. (TIF) [file pone.0125980.s001.tif]

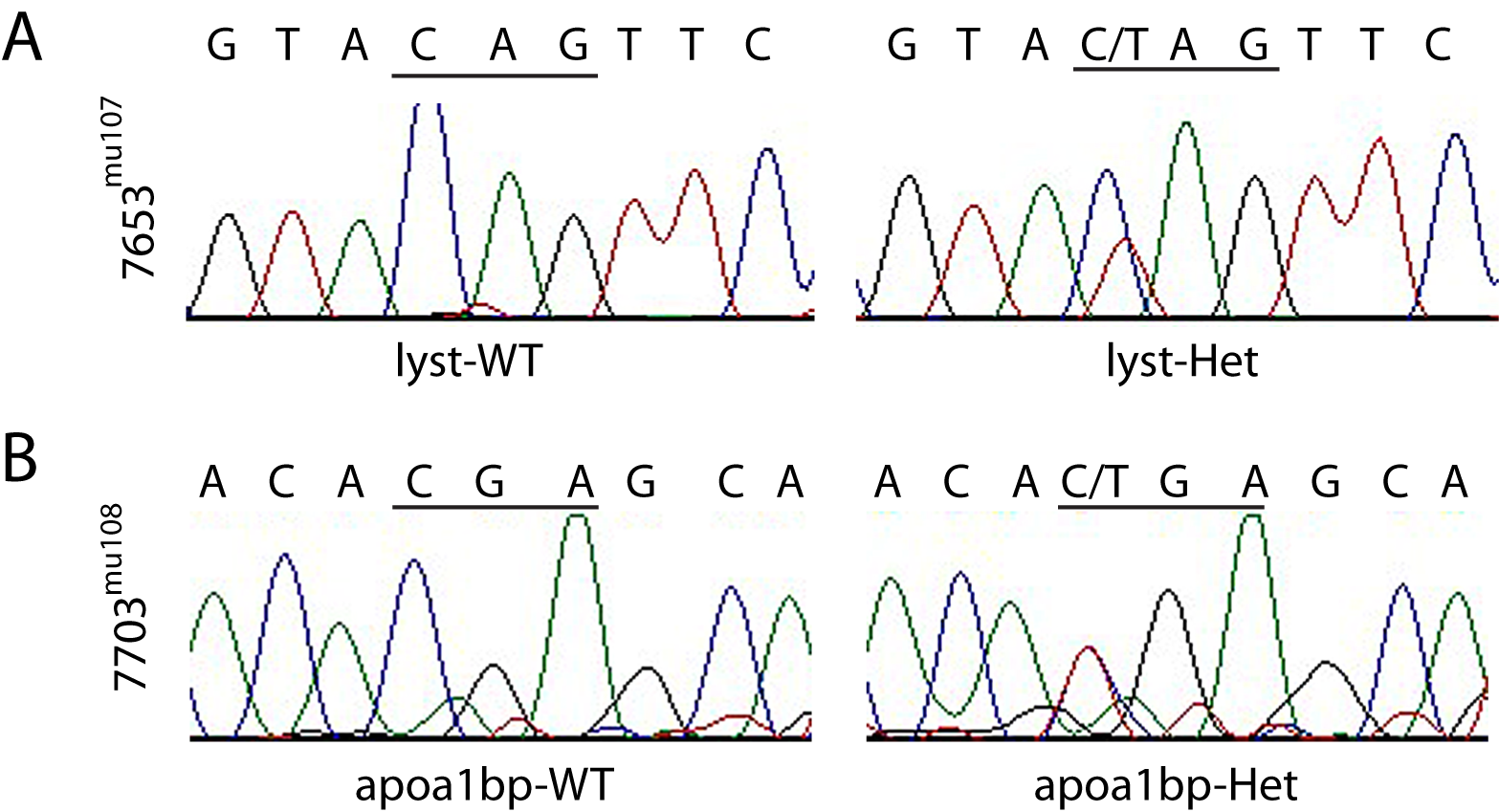

Supplement: S2 Fig — null mutations in lyst (A) and apoa1bp (B) were identified by whole genome sequencing and both mutations were confirmed in heterozygous mutant adult. C to T conversion caused Q336 to stop in lyst (A) and R26 to stop in apoa1bp (B). (TIF) [file pone.0125980.s002.tif]
